# Mechanistic investigation of capability of enzymatically synthesized polycysteine to cross-link proteins

**DOI:** 10.1016/j.bbrep.2016.07.013

**Published:** 2016-07-21

**Authors:** Asako Narai-Kanayama, Tomoko Hanaishi, Keiichi Aso

**Affiliations:** Faculty of Applied Life Science, Nippon Veterinary and Life Science University, 1-7-1 Kyonan-cho, Musashino-shi, Tokyo 180-8602, Japan

**Keywords:** Cys-OEt, l-cysteine ethyl ester, DMSO, dimethyl sulfoxide, DP, degree of polymerization, DTNB, 5,5′-dithiobis (2-nitrobenzoic acid), DTT, dithiothreitol, IAM, iodoacetamide, MALDI-TOF MS, matrix assisted laser deso′rption/ionization time of flight mass spectrometry, PLCys, poly-l-cysteine, *S*-CM, S-carbamoylmethyl, SEC, size exclusion chromatography, Poly-l-cysteine, Lysozyme, Disulfide bond, SH/SS exchange, Protein cross-linking

## Abstract

**Background:**

Previously, we had reported that α-chymotrypsin–catalyzed polymerization of l-cysteine ethyl ester in a frozen buffer provided poly-l-cysteine (PLCys) in good yield, of which degree of polymerization had been determined to be 6–11. Almost all of SH groups in PLCys were in free forms. Such a multi-thiol peptide may cross-link proteins through thiol/disulfide (SH/SS) exchange reactions, considering the knowledge that other synthetic multi-thiol additives changes properties of protein materials.

**Methods:**

This study explored the capability of PLCys to cross-link proteins using lysozyme as a model protein which has four disulfide bonds but no free SH group. The protein was incubated with PLCys at neutral pH and at below 70 °C to avoid PLCys-independent, β-elimination-mediated cross-linkings. Protein polymerization was analyzed by SDS-PAGE and SEC. PLCys peptides involved in the protein polymer, which were released by reduction with dithiothreitol, were analyzed by RP-HPLC.

**Conclusions:**

Addition of urea and thermal treatment at 60 °C caused PLCys-induced lysozyme polymerization. Compared with free cysteine, a higher level of PLCys was required for the polymerization probably due to its low water solubility. RP-HPLC analyses suggested that PLCys played a role in the protein polymerization as a cross-linker.

**General significance:**

Enzymatically synthesized PLCys shows promise as a peptidic cross-linker for the production of protein polymers with novel physiochemical properties and functionalities.

## 1 Introduction

Protein cross-linking plays an important role in the functionality of food and non-food proteins. Cross-linking of proteins leads to the creation of macromolecular assemblies with new or modified physicochemical properties and functionalities [1]. Recently, research into enzymatic cross-linking has become prominent in many fields including food technology as well as biochemical and biomedical research [2], [3], [4], [5]. For example, the transglutaminase-catalyzed formation of 3-dimensional protein networks is based on the condensation of Gln and Lys residues by transamidation [6], [7]. Indeed, the application of transglutaminase in food processing has been reported to improve the technical characteristics of proteins, such as gel formation as well as emulsifying and rheological properties [2], [3].

Conventionally pre-existing disulfide (SS) bonds also contribute to protein cross-linking. In contrast to the irreversible covalent bonds found in other types of protein linkages, the SS bonds can be reversibly cleaved by reducing agents. In addition, the reshuffling reaction between thiol (SH) groups and intra- and intermolecular SS bonds occurs with thermal treatment of protein solutions [8]. Not only this reaction but also disulfide formation are pH-dependent [8], [9]. Many researchers have characterized the relationship between the SS bonds in gluten and the extensibility and elasticity of wheat dough [10], [11], [12], [13]. Thermal treatment of gluten consisting of monomeric gliadin and polymeric glutenin induces intra- and intermolecular SH/SS exchange reactions, resulting in the incorporation of gliadin into the glutenin network. The structure, density, and strength of the gluten network affect the property of dough, which determines the bread quality. Thus, the effects of redox agents on gluten have been studied [14], [15], [16]. It has also been reported that the addition of free Cys into a gliadin solution followed by gentle stirring causes polymerization of the protein even at 40 °C for 30 min [17]. Protein films derived from Cys-mediated gliadin polymerization show water vapor permeability comparable to glutenin films. Such films made from cross-linked food proteins have received considerable attention as a promising material for use in food packaging and agricultural applications.

We recently reported that α-chymotrypsin-catalyzed polymerization of L-cysteine ethyl ester (Cys-OEt) in a frozen buffer solution produces water-insoluble poly-L-cysteine (PLCys), reaching 85% yield on a substrate basis [18]. One advantage of this synthetic method is its simplicity, not requiring blocking/deblocking or any harmful reagents. The degree of polymerization (DP) of the synthesized PLCys was from 6 to 11 as determined using MALDI-TOF MS. Most of the SH groups in PLCys were found to be in the free form. Recently, other researchers also reported that papain- and proteinase K-catalyzed synthesis of PLCys from Cys-OEt can be successfully accomplished at 40 °C [19], [20]. Furthermore, Ma *et al.* demonstrated that PLCys in the solid state is stable at broad temperatures up to 200 °C [20]. If PLCys can join the intermolecular SH/SS exchange together with proteins, it would work as a cross-linker providing protein polymers with novel physicochemical properties and functionalities.

In the present study, we investigated whether PLCys could help protein cross-linking through SH/SS exchange and function as a cross-linker. To focus on the effects of SH groups in PLCys, lysozyme was chosen as a model protein, which has four SS bonds but no free SH group [21], [22]. In addition, its molecular size of 14.3 kDa makes it facile to determine polymerization of the protein by polyacrylamide gel electrophoresis.

## 2 Materials and methods

### 2.1 Materials

l-Cysteine ethyl ester hydrochloride was purchased from Tokyo Kasei Co. (Tokyo, Japan). α-Chymotrypsin (EC 3.4.21.1) type II from bovine pancreas, 3×crystallized from 4× crystallized chymotrypsinogen, dialyzed essentially salt-free and prepared as lyophilized powder, which was 54 U/mg protein determined at 25 °C and pH 7.8 with *N*-benzoyl-l-tyrosine ethyl ester as a substrate, lysozyme from chicken egg white, l-arginine ethyl ester dihydrochloride and ProteoMass™ peptide MALDI-MS calibration kit were purchased from Sigma-Aldrich Co. (St. Louis, MO, USA). 2,5-Dihydroxybenzoic acid, DMSO, trifluoroacetic acid (TFA) and urea were purchased from Kanto Chemical Co. (Tokyo, Japan). Iodoacetamide (IAM) and 5,5-dithiobis (2-nitrobenzoic acid) (DTNB) were obtained from Nacalai Tesque Inc. (Kyoto, Japan). 2,4,6-Trinitrobenzenesulfonic acid sodium salt dihydrate was obtained from Wako Pure Chemical Industries Ltd. (Osaka, Japan). Standards for size exclusion chromatography were from Bio-Rad (Hercules, CA, USA). All other regents used were of analytical grade.

### 2.2 Enzymatic polymerization of Cys-OEt and preparation of PLCys

Conditions for enzymatic polymerization of Cys-OEt were as follows: 100 mM Cys-OEt and 20 μM α-chymotrypsin were mixed in 100 mM Na-phosphate buffer (pH 8.0), and then the mixture was immediately frozen at −20 °C and stayed for 3 days. To stop the reaction, 1/5 vol of 5 N HCl was added into the frozen reaction mixture and then it was thawed, preventing its temperature from reaching 10 °C. The precipitated PLCys was collected, rinced and dried as described previously [18]. It was kept in the dark at 4–8 °C until before use for various analyses. Quantitative assays of the DTNB-reactive SH groups in PLCys were performed as described previously [18]. After PLCys was dissolved in DMSO, about 65% of Cys residues in PLCys could be determined using DTNB [18]. Based on this, theoretical Cys residues in PLCys were estimated.

### 2.3 Reaction of PLCys with lysozyme

Prepared PLCys was suspended in 200 mM Na-phosphate, pH 7.0, so as to contain DTNB-reactive SH groups of 21 mM (theoretical Cys residues: 33 mM). This suspension was mixed with 6 mg/mL (about 0.4 mM) of lysozyme in pure water with or without 8 M urea in a 1:1 vol ratio, each of their final concentrations becoming half. The mixture was incubated for 30 min at 30 or 60 °C. After the reaction mixture was cooled on ice for 5 min, it was mixed with 1/5 vol of 200 mM IAM in 100 mM Na-phosphate buffer (pH 8.0), and then the pH was adjusted to 8.0 by the addition of 1 N NaOH. It was incubated for 1 h at room temperature in the dark to modify remaining free SH groups. The sample was centrifuged at 15,000×*g* for 30 min at 25 °C. The supernatant was analyzed by SDS-PAGE, SEC, and ultrafiltration followed by RP-HPLC.

### 2.4 Ultrafiltration of lysozyme-PLCys reaction mixture

The supernatant of the lysozyme-PLCys reaction mixture was loaded onto the membrane filter of an Amicon Ultra centrifugal filter unit of 30 kDa molecular weight cut off (Millipore Ireland Ltd. Cork, Ireland). To prevent aggregation of proteins, l-arginine ethyl ester dihydrochloride (at final concentration of approx. 1 M) was added into the sample [23]. After centrifugation at 5000×*g* at 25 °C, the ultrafiltrate was transferred to an Amicon Ultra filter of 10 kDa for further ultrafiltration. Each of the higher molecular weight (>30 kDa) and 10–30 kDa fractions was rinsed with 4 M urea/0.05 N HCl (pH 4.0) five times, and then, was concentrated by about 30-fold in the ultrafiltration device. Such a mild acidic conditions was used to inhibit β-elimination of SS bonds.

### 2.5 SDS-PAGE

The supernatant of lysozyme-PLCys (or Cys) reaction mixture was analyzed by SDS-PAGE [24] with a separating gel of 12.5% polyacrylamide. Prior to electrophoresis, the sample was mixed with an equal volume of 60 mM Tris-HCl (pH 7.4) containing 2% SDS, 20% glycerol, and 0.02% bromophenol blue with or without 1.5% DTT, and then incubated for 1 h at room temperature in the dark. Twenty microliters of each sample was loaded. Gels were stained by Coomassie Brilliant Blue (CBB) R-250.

### 2.6 Size exclusion chromatography (SEC)

The supernatant of the lysozyme-PLCys (or Cys) reaction mixture was filtered through a 0.45 µm filter (DISMIC-13cp, Advantec Toyo, Tokyo, Japan), and then, the filtrate was loaded (20 μL) on a Showdex KW 803 column (Showa Denko, Tokyo, Japan). The proteins were eluted at room temperature with 2% SDS at flow rate of 1 mL/min using a Hitachi L-2700 pump, and detected at 280 nm by an L-2400 UV detector. Chromatograms were analyzed using the data processing software Chromato-PRO (Run Time Corporation, Kanagawa, Japan). Proteins were classified into (1) monomers of about 14 kDa and (2) polymers of broad molecular sizes from 28 kDa to 110 kDa. The proportion of lysozyme in these two groups was estimated from peak areas, based on the peak area of intact (control) lysozyme as 100%.

### 2.7 RP-HPLC

For investigation of PLCys involved in the polymerized lysozyme complex, each sample was mixed with an equal volume of 0.1 M DTT in 50 mM Na-phosphate (pH 8.0) containing 4 M urea, and then incubated for 1 h at room temperature in the dark. The sample was filtered through a 0.45 µm filter, and then, an aliquot (50 μL) was analyzed using a Hitachi HPLC system (L-2700 pump, L-2400 UV detector) equipped with an Inertsil WP300 C8 (5 µm, 4.6 mm×150 mm) column (GL Science, Tokyo, Japan). The mobile phase used was a gradient of 0–16–72–80–80% CH_3_CN/0.1% TFA in 0–30-54–55–65 min at a flow rate of 1.0 mL/min. Intact PLCys of different DP, each of which has completely free SH groups, and S-carbamoylmethylated (*S*-CM) PLCys were separately eluted and detected at 220 nm. To confirm the retention time of intact PLCys, prepared PLCys was dissolved in DMSO and then injected to the HPLC. The S-carbamoylmethylation of intact PLCys was conducted by adding 20 mM IAM in 50 mM Tris-HCl (pH 9.0) into the PLCys/DMSO solution containing approx. 5 mM of DTNB-reactive SH groups (theoretical Cys residue: 7.7 mM) in a volume ratio of 1:1. After a 30-min reaction at room temperature in the dark, the *S*-CM PLCys peptides were analyzed by RP-HPLC as described above.

### 2.8 MALDI-TOFMS analysis

MALDI mass spectral data were acquired using a KOMPACT/SEQ V1.2.2 (Shimadzu Co., Kyoto, Japan) operating in positive ion mode. The sample purified through RP-HPLC was mixed with 2,5-dihydroxybenzoic acid as a matrix and then loaded on a sample plate. Analysis was performed as described previously [25].

## 3 Results

### 3.1 Interaction of Cys or PLCys with lysozyme at neutral pH

We confirmed that lysozyme self-aggregated and precipitated at pH 7.0 at above 70 °C (Suppl. 1). This was similar to thaumatin, a sweetener protein that has eight SS bonds but no free Cys residues, which was reported to self-aggregate upon heating at pH 7.0 at above 70 °C through β-elimination of SS bonds [26]. Therefore, the effects of PLCys on lysozyme were investigated at below 70 °C, 30 °C or 60 °C, in order to avoid PLCys-independent protein cross-linking. Lysozyme of 3 mg/mL (0.2 mM) was incubated with Cys or PLCys containing 10.5 mM of DTNB-reactive SH (theoretical Cys residues in PLCys: 16 mM) in 100 mM Na-phosphate (pH 7.0) for 30 min. Since a molecule of lysozyme has four SS bonds, the Cys residues derived from PLCys were estimated to be ten times as much as those of the protein. After the remaining SH groups were modified by IAM, each sample was analyzed by SDS-PAGE.

**Fig. 1:**
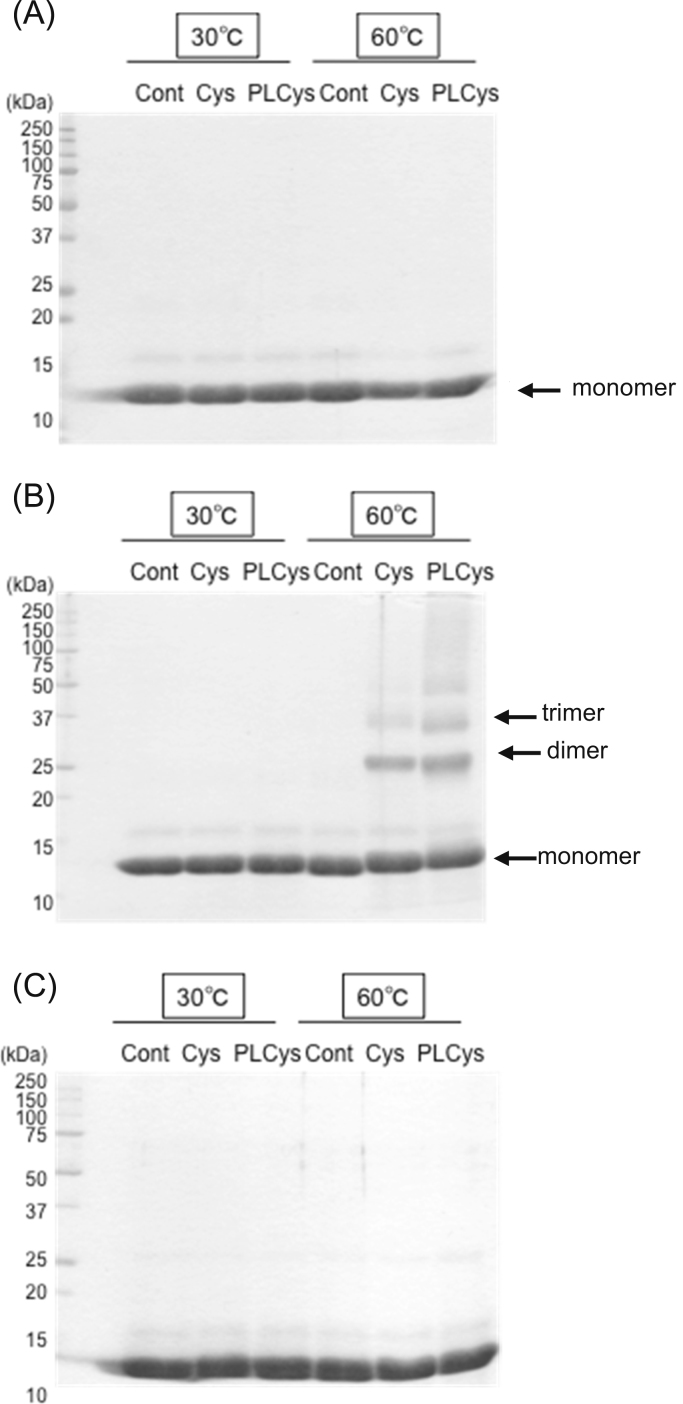
Non-reducing SDS-PAGE of the supernatant lysozyme solution without (A) or with 4 M urea (B) after incubation with Cys or PLCys at pH 7.0 and reducing SDS-PAGE (C) of the same samples as (B). Lysozyme of 3 mg/mL in 100 mM Na-phosphate (pH 7.0) was incubated for 30 min without or with thiol compounds of 10.5 mM DTNB-reactive SH groups.

There was no change in lysozyme in normal phosphate buffer solution after incubation with or without Cys and PLCys both at 30 °C and 60 °C (Fig. 1A). However, the addition of urea into the sample solution caused the Cys- or PLCys-induced polymerization of lysozyme at 60 °C (Fig. 1B). In order to investigate reducing effects on the protein polymers in the soluble fraction, the lysozyme samples were treated with β-mercaptoethanol at 3% (w/v) or DTT at 1.5% (w/v) for 1 h, and then loaded on SDS-PAGE. Most of the protein polymers were reduced by DTT, providing monomers (Suppl. 2, Fig. 1C). However, slightly more dimers remained in samples treated with β-mercaptoethanol even at higher molar concentration of SH group than that in DTT (see Suppl. 2). Thus, we chose DTT rather than β-mercaptoethanol in this study to prevent incomplete reduction of protein polymers.

**Fig. 2:**
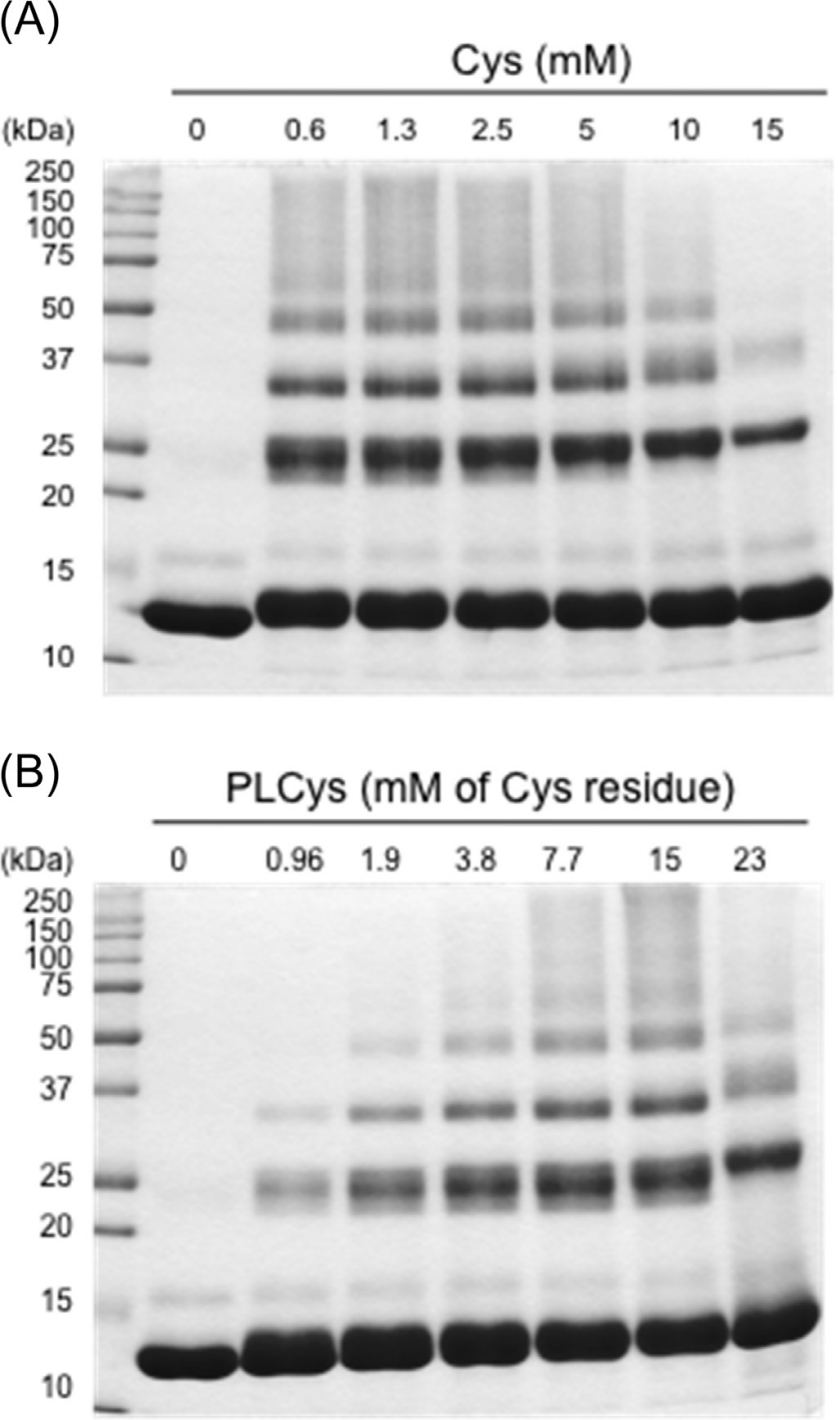
Effects of concentrations of Cys and PLCys on polymerization of lysozyme. Lysozyme of 3 mg/mL in 100 mM Na-phosphate (pH 7.0) containing 4 M urea was incubated with Cys or PLCys at 60 °C for 30 min. The supernatants were analyzed by non-reducing SDS-PAGE.

**Fig. 3:**
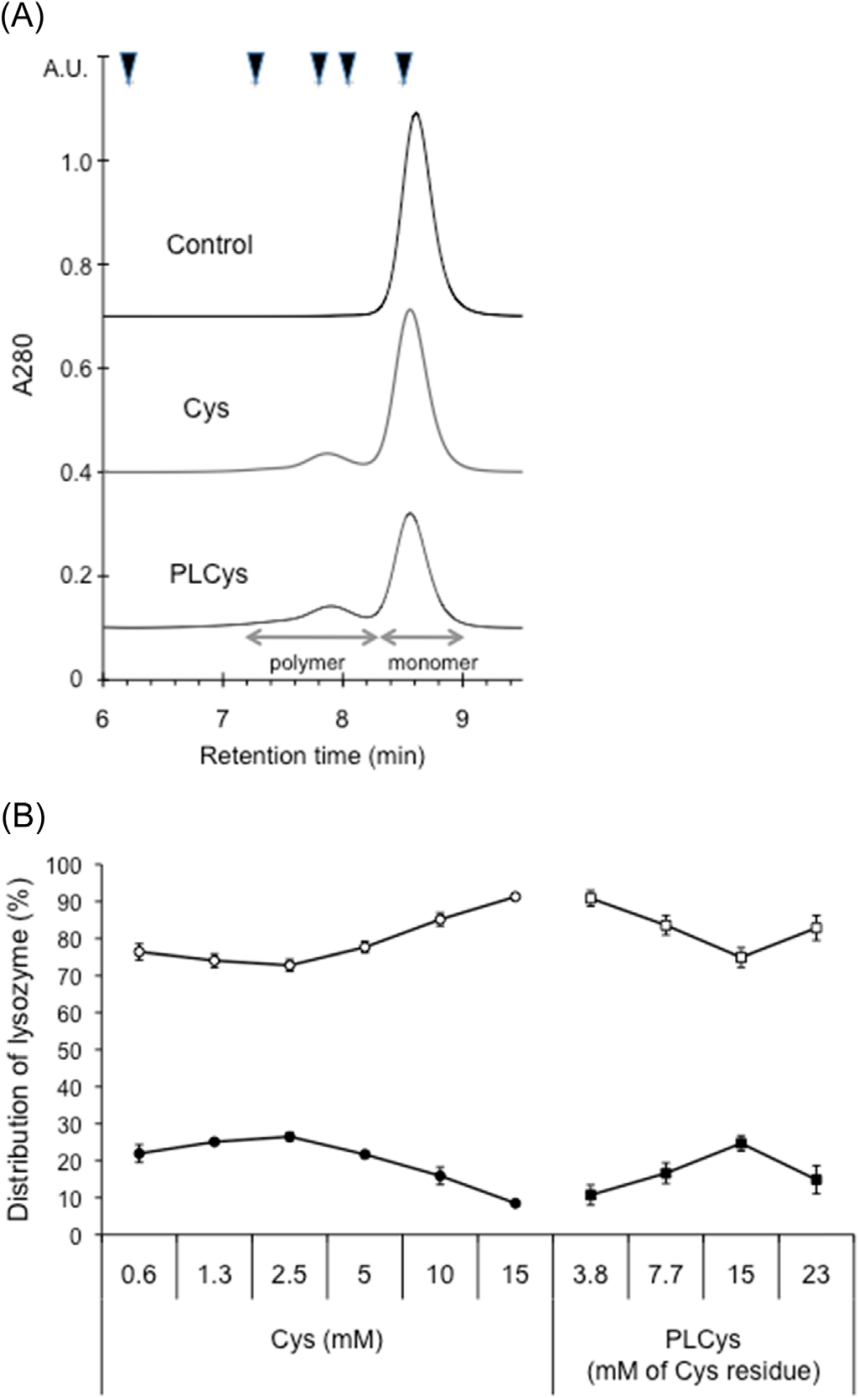
Changes in SEC chromatograms of lysozyme incubated with Cys or PLCys (A) and distribution of lysozyme to monomer and polymers (B). Lysozyme of 3 mg/mL in 100 mM Na-phosphate (pH 7.0) containing 4 M urea was incubated with Cys or PLCys at 60 °C for 30 min. The supernatants (each 20 μL) were loaded to SEC. (A) Chromatograms of control, Cys, and PLCys were overlaid with offset of 60 mA.U. The arrowheads indicate retention times of molecular standards (thyroglobulin (bovine): 670 kDa, γ-globulin (bovine): 158 kDa, serum albumin (bovine): 66 kDa, ovalbumin (chicken): 44 kDa, and myoglobin (horse): 17 kDa). (B) The area proportions of lysozyme monomer (open symbol) and polymers (closed symbol) are indicated as % of the control (means ± SD, n=3).

**Fig. 4:**
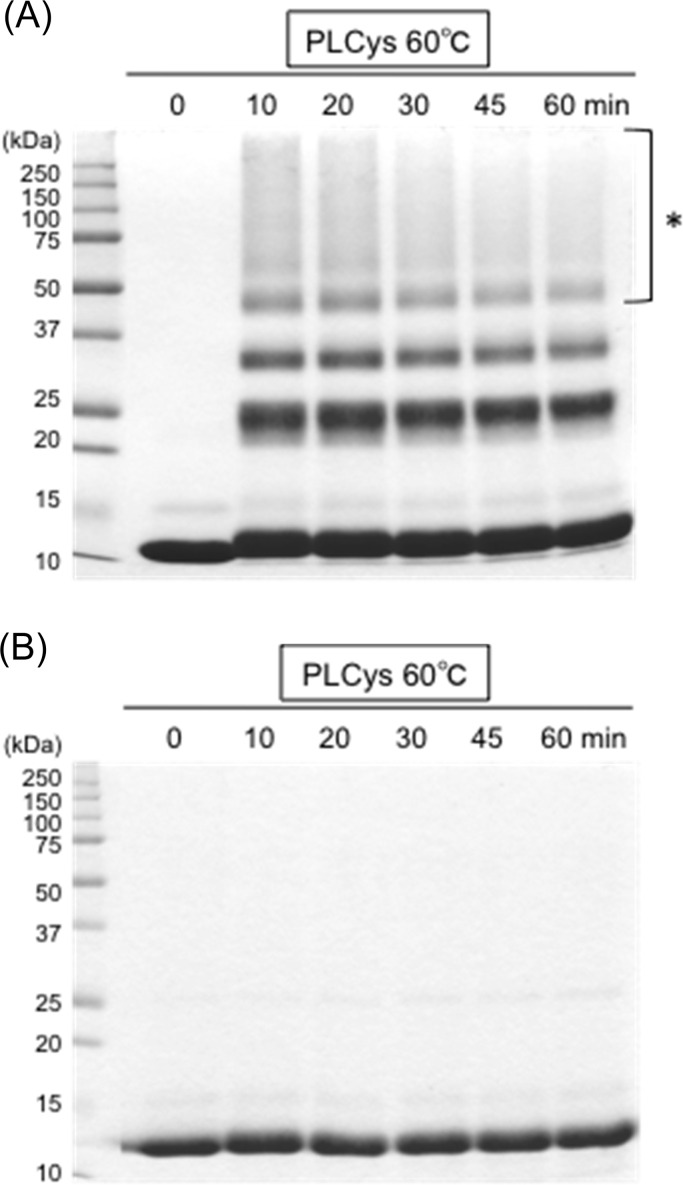
Analyses of temporal change of lysozyme during incubation with urea and PLCys at pH 7 by non-reducing (A) and reducing (B) SDS-PAGE. Lysozyme of 3 mg/mL in 100 mM Na-phosphate (pH 7.0) containing 4 M urea was incubated with PLCys containing Cys residue of 8 mM. The supernatants were analyzed. The synthesized polymers decreased as the incubation was prolonged (asterisk).

The fact that urea and heating slightly increased the amount of PLCys in the soluble fraction (Table 1) might have resulted in the acceleration of intermolecular SH/SS exchange between PLCys and lysozyme. However, the Cys- and PLCys-induced polymerization of lysozyme was not simply correlated to their concentrations (Fig. 2, Fig. 3). For Cys, low SH levels were enough to cause protein polymerization while high levels restricted it (Figs. 2A and 3). That was true for PLCys. Temporal changes in the behavior of lysozyme during the incubation with PLCys for 60 min at 60 °C indicated that the previously synthesized polymers decreased as the incubation was prolonged (asterisk in Fig. 4A). Almost all of the polymers were reduced by treatment with DTT (Fig. 4B). These results suggested that excessive amounts of SH groups cleaved the SS bonds related to the protein polymerization.

**Table 1:** Effects of urea and heating on the solubility of PLCys.

	Control		4 M urea	
SH groups in supernatant	30 °C	60 °C	30 °C	60 °C
	8.2 ± 1.1%	14.0 ± 2.0%⁎	13.2 ± 0.5%⁎	20.7 ± 2.1%⁎

Prepared intact PLCys was suspended in 0.2 M Na-phosphate buffer (pH 7.0) with or without 4 M urea. The sample was incubated at 30 °C or 60 °C for 30 min, and then centrifuged at 15,000×*g* at 25 °C for 30 min. The SH contents of the supernatant were determined by DTNB. Data are indicated as mean ± SD (n=4, 100%= prepared intact PLCys).

⁎ Significantly different from the supernatant after incubation at 30 °C without urea (p<0.05).

### 3.2 Analysis of PLCys associated with lysozyme

**Fig. 5:**
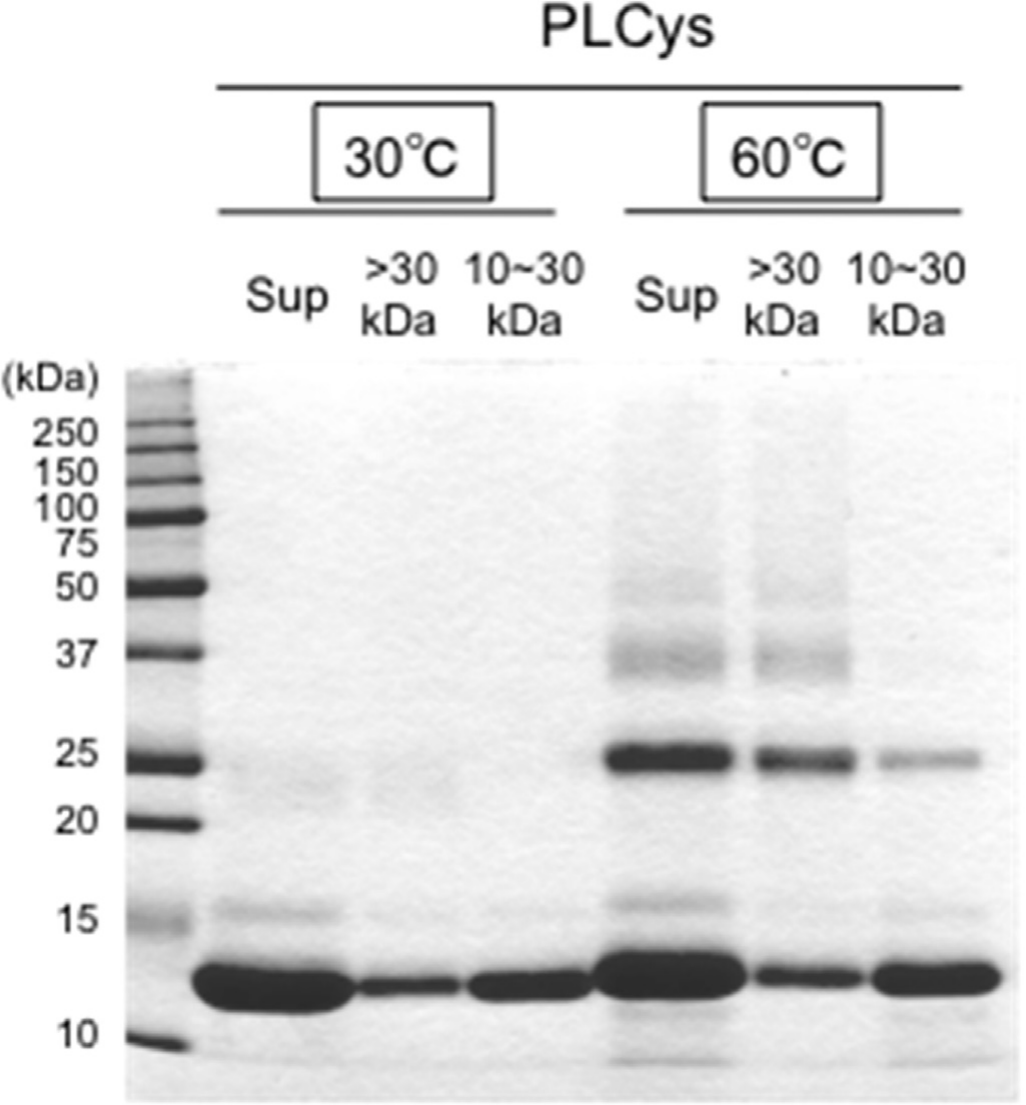
Size-fractionated lysozyme and its polymers. Lysozyme of 3 mg/mL in 100 mM Na-phosphate (pH 7.0) containing 4 M urea was incubated without (control) or with PLCys at 60 °C for 30 min. Proteins in the supernatant (Sup) were size-fractionated into >30 kDa and 10–30 kDa fractions by ultrafiltration, and then were analyzed by non-reducing SDS-PAGE.

**Fig. 6:**
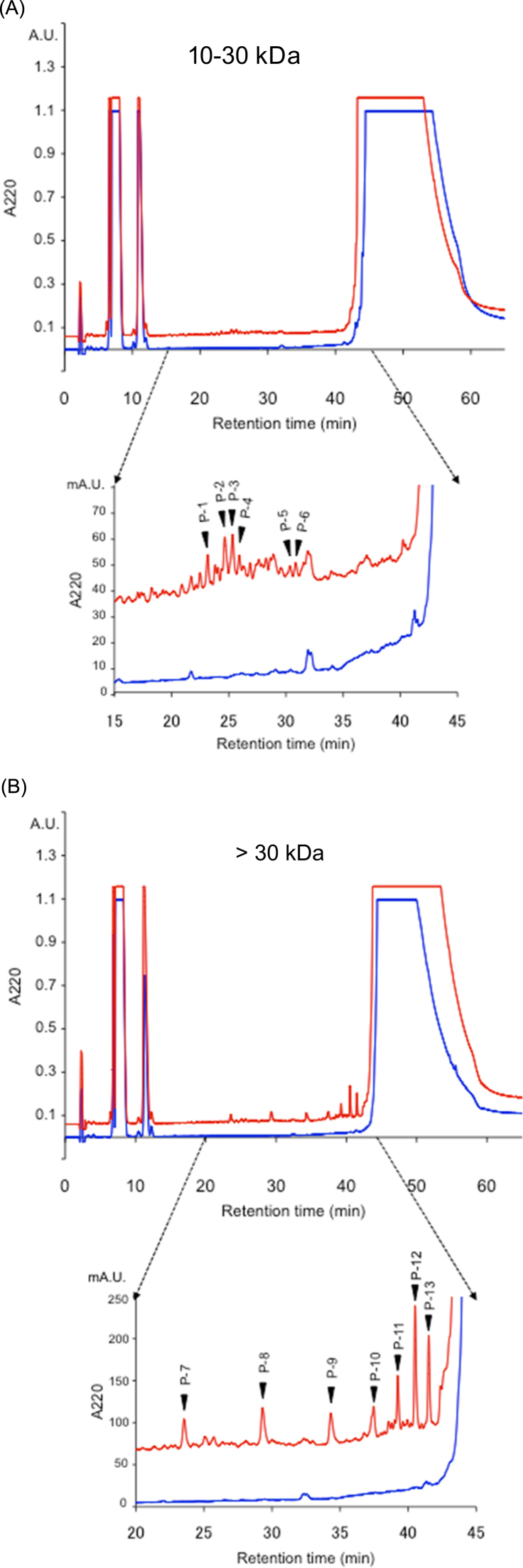
RP-HPLC analyses of PLCys involved in lysozyme polymers. After the 10–30 kDa (A) and >30 kDa (B) fractions were reduced with DTT, each was analyzed (lower line: control, upper line: PLCys; these two chromatograms were overlaid with offset of 60 mA.U.). The large peak eluted after 40 min was lysozyme.

After the incubation of lysozyme with PLCys at 60 °C followed by treatment with IAM, the sample solution was size-fractionated into two fractions, >30 kDa and 10–30 kDa, by ultrafiltration. The polymerized proteins in the higher molecular weight fraction aggregated during concentration by ultrafiltration without adding anything, probably due to non-specific and non-covalent binding such as hydrogen bonds and hydrophobic interactions. However, the addition of l-arginine ethyl ester into the sample solution effectively inhibited the aggregation [23]. The >30 kDa fraction contained lysozyme dimer, trimer, and further polymers, while most of the monomers and a small amount of dimers were obtained in the 10–30 kDa fraction (Fig. 5). Each of the two fractions was treated with DTT, and then an aliquot was loaded on a C8 column for RP-HPLC analysis. Compared with the control, reduction of the PLCys-treated sample released some peptides shown as the peaks P-1 to P-13 in Fig. 6A and B.

**Fig. 7:**
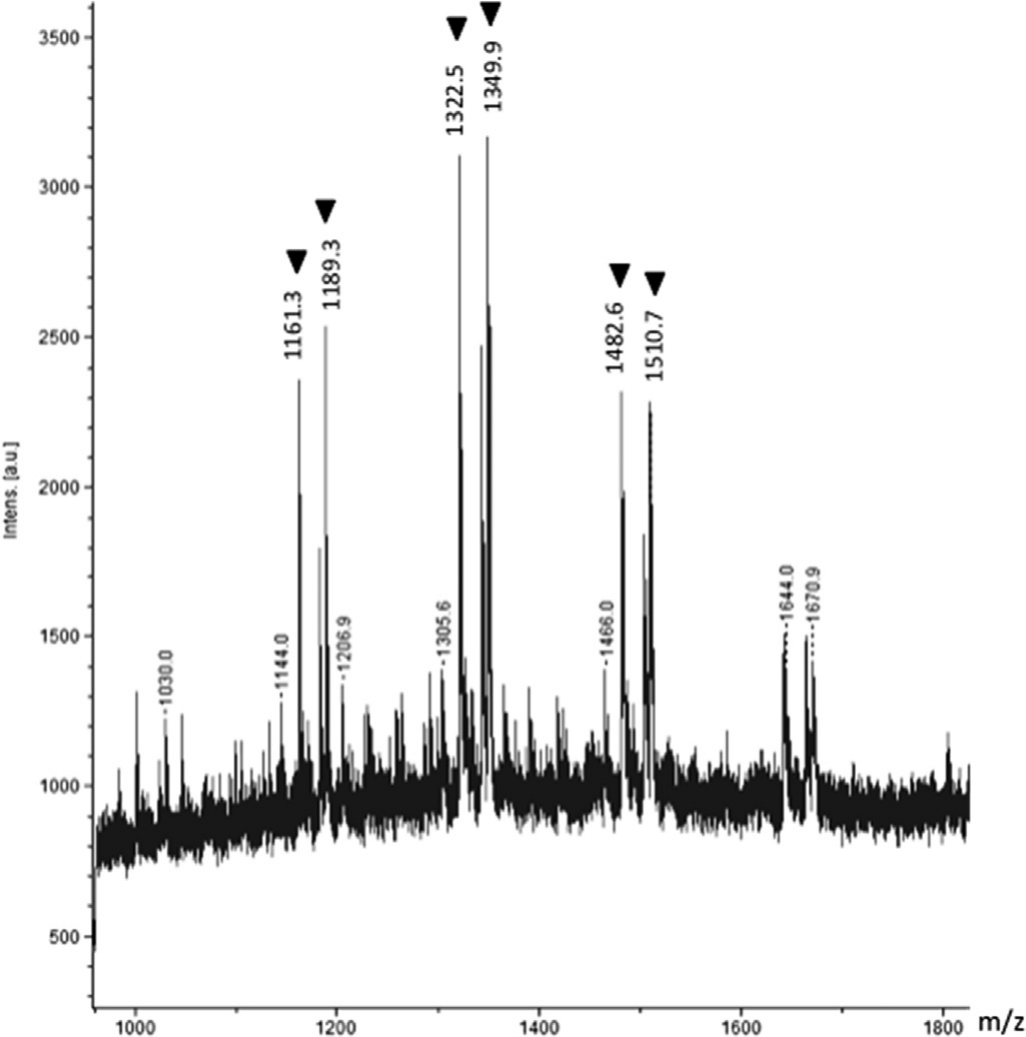
Mass spectrum of PLCys treated with IAM after incubation with 4 M urea, at 60 °C for 30 min. Detected *m/z* signals indicated by arrowheads were identified as [*S*-CM PLCys peptides +Na^+^] ions, of which theoretical *m/z* values were 1161.2 (DP7,-OH, 7×*S*-CM), 1189.1 (DP7, ethyl ester (-OEt), 7×*S*-CM), 1321.2 (DP8,-OH, 8×*S*-CM), 1349.8 (DP8,-OEt, 8×*S*-CM), 1481.3 (DP9,-OH, 9×*S*-CM), and 1509.8 (DP9,-OEt, 9×*S*-CM).

**Fig. 8:**
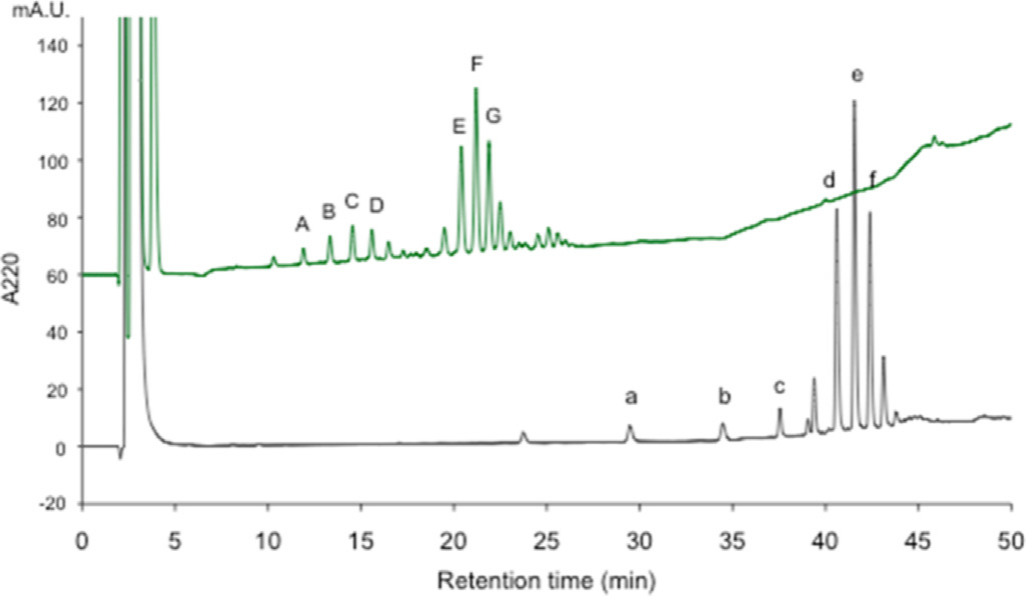
RP-HPLC analyses of intact PLCys (lower) and *S*-CM PLCys (upper). Prepared intact PLCys was dissolved in DMSO. This PLCys/DMSO sample was treated with IAM for S-carbamoylmethylation. Two chromatograms were overlaid with offset of 60 mA.U.

In contrast, the supernatant of only the PLCys sample without lysozyme, which was incubated at 60 °C with urea, did not give any peaks even after a series of treatments (S-carbamoylmethylation, ultrafiltration, and reduction). MALDI-TOF MS analysis of the sample after S-alkylation showed weak *m/z* signals (Fig. 7). They were identified as completely S-carbamoylmethylated (*S*-CM) PLCys peptides, suggesting that PLCys did not self-aggregate through formation of the inter- and intra-molecular SS bonds under the conditions in this study. We also confirmed that SH groups in the PLCys dissolved in DMSO were completely modified by IAM (Fig. 8 and Table 2, Table 3). These results indicated that all SH groups in PLCys, once solubilized and/or present in media, could be modified by IAM without steric hindrance.

**Table 2:** Identification of *S*-CM PLCys using MALDI-TOF MS.

PLCys (DP, C-terminal form⁎, number of *S-*CM group, added ion)	Calculated *m/z*	Corresponding HPLC peaks (detected *m/z*)
[DP6, -OH, 6*S*-CM, H^+^]	979.2	A (980.6, 1001.8)
[DP6, -OH, 6*S*-CM, Na^+^]	1001.2	
[DP7, -OH, 7*S*-CM, H^+^]	1139.2	B (1140.9, 1162.1)
[DP7, -OH, 7*S*-CM, Na^+^]	1161.2	
[DP8, -OH, 8*S*-CM, H^+^]	1299.3	C (1300.2, 1321.9)
[DP8, -OH, 8*S*-CM, Na^+^]	1321.2	
[DP9, -OH, 9*S*-CM, H^+^]	1459.3	D (1458.7, 1481.4)
[DP9, -OH, 9*S*-CM, Na^+^]	1481.3	
[DP7, -OEt, 7*S*-CM, H^+^]	1167.3	E (1167.4, 1189.1)
[DP7, -OEt, 7*S*-CM, Na^+^]	1189.1	
[DP8, -OEt, 8*S*-CM, H^+^]	1328.0	F (1328.0, 1349.8)
[DP8, -OEt, 8*S*-CM, Na^+^]	1349.8	
[DP9, -OEt, 9*S*-CM, H^+^]	1488.6	G (1488.6, 1509.8)
[DP9, -OEt, 9*S*-CM, Na^+^]	1509.8	

⁎ -OH: carboxy group, -OEt: ethyl ester group.

**Table 3:** Identification of intact PLCys and PLCys released from the >30 kDa fraction using MALDI-TOF MS.

PLCys (DP, C-terminal form, added ion)	Calculated *m/z*	Corresponding HPLC peaks (detected *m/z*)	
[DP5, -OH, H^+^]	534.1	P-7 (534.8, 556.3)	–
[DP5, -OH, Na^+^]	556.0		
[DP6, -OH, H^+^]	637.1	P-8 (638.4, 658.6)	a (637.4, 659.1)
[DP6, -OH, Na^+^]	659.1		
[DP7, -OH, H^+^]	740.1	P-9 (740.8, 762.2)	b (740.4, 762.0)
[DP7, -OH, Na^+^]	762.1		
[DP8, -OH, H^+^]	843.1	–	c (865.0, 893.1)
[DP8, -OH, Na^+^]	865.1		
[DP5, -OEt, H^+^]	562.1	P-10 (561.8, 584.0)	–
[DP5, -OEt, Na^+^]	584.1		
[DP6, -OEt, H^+^]	665.1	P-11 (665.0, 686.8)	–
[DP6, -OEt, Na^+^]	687.1		
[DP7, -OEt, H^+^]	768.1	P-12 (768.2, 789.8)	d (768.1, 790.1)
[DP7, -OEt, Na^+^]	790.1		
[DP8, -OEt, H^+^]	871.1	P-13 (871.2, 893.8)	e (871.1, 893.1)
[DP8, -OEt, Na^+^]	893.1		
[DP9, -OEt, H^+^]	974.1	–	f (996.2)
[DP9, -OEt, Na^+^]	996.1		

**Scheme 1:**
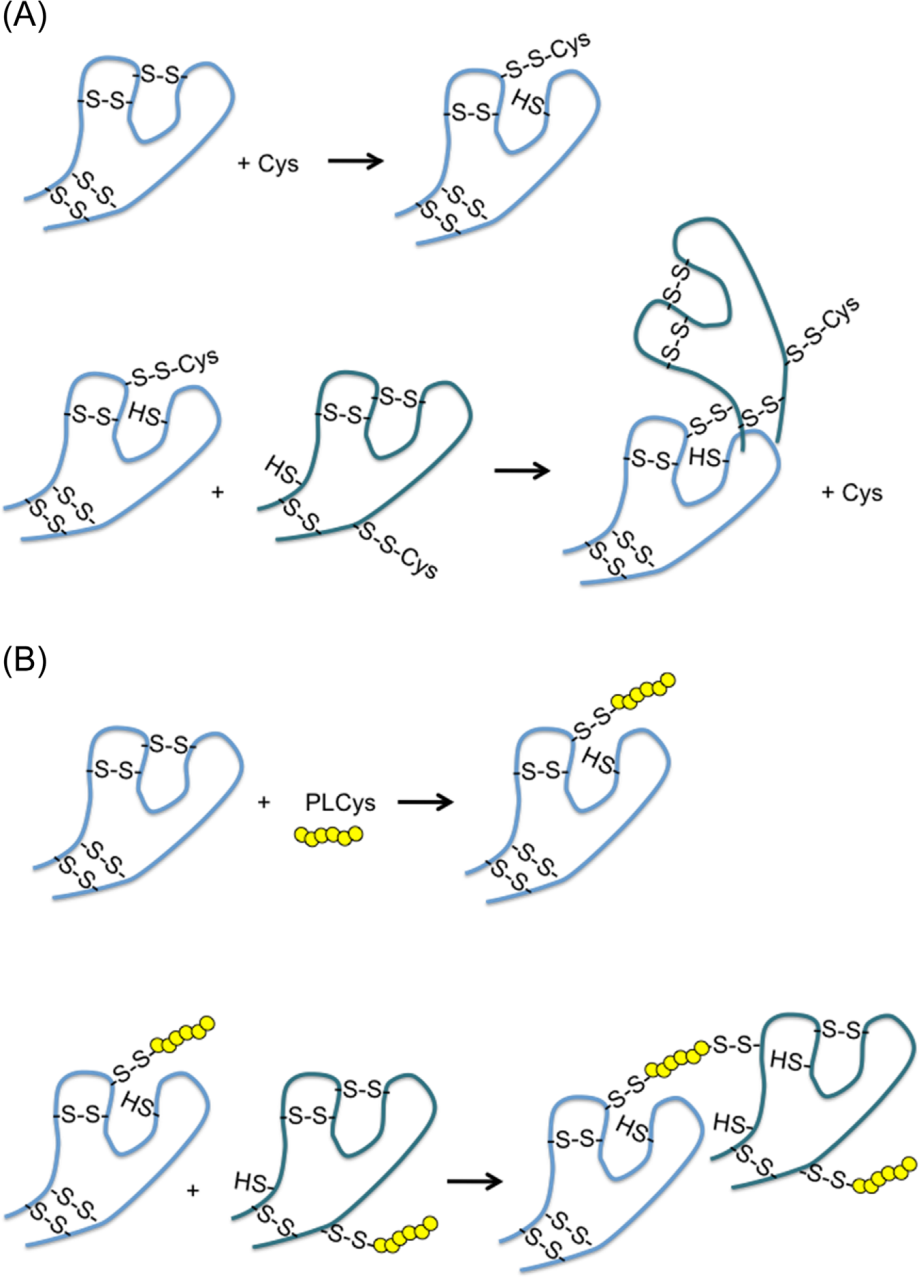
Possible mechanism of lysozyme polymerization by Cys (A) and PLCys (B). -SH and S-S indicate thiol group and disulfide bond, respectively.

By MALDI-TOF MS analysis, we found that P-1 to P-6 derived from the 10–30 kDa fraction corresponded to the partially S-carbamoylmethylated PLCys peptides (Table 4). We assumed that the SH/SS exchange reactions occurring between Cys and lysozyme molecules resulted in the formation of tightly associated polymers (Scheme 1A). Meanwhile, PLCys might contribute to the polymerization of lysozyme as a cross-linker (Scheme 1B). Since PLCys has several free SH groups, many kinds of patterns of SH/SS exchange are possible. The *S*-CM PLCys detected as P-1 to P-4 indicated that one of the Cys residues in PLCys bound to the protein through SH/SS exchange as depicted in Scheme 1B. The resulting appearance of latent SH on the protein could trigger further intra- and intermolecular SH/SS exchange reactions. P-5 and P-6 suggested that the other remaining SH groups in PLCys also participated in the formation of intra- or intermolecular SS bonds. P-7 to P-13 derived from the >30 kDa fraction proved to be intact molecules of PLCys (Table 3). The release of intact PLCys peptides from the >30 kDa fraction suggested that PLCys was involved in the highly polymerized protein as a cross-linker. Such PLCys peptides might bind to proteins by multiple SS bonds, so they could not interact with IAM probably due to steric hindrance as well as the complete absence of free SH groups.

**Table 4:** Identification of *S*-CM PLCys released from the 10–30 kDa fraction using MALDI-TOF MS.

PLCys (DP, C-terminal form, number of *S-*CM group, added ion)	Calculated *m/z*	Corresponding HPLC peaks (detected *m/z*)
[DP6, -OEt, 5*S*-CM, Na^+^]	972.2	P-1(971.6)
[DP7, -OEt, 6*S*-CM, H^+^]	1110.2	P-1(1109.6), P-2(1109.7)
[DP7, -OEt, 6*S*-CM, Na^+^]	1132.2	P-1(1132.3)
[DP8, -OEt, 7*S*-CM, H^+^]	1270.3	P-1(1270.8), P-3(1270.8)
[DP8, -OEt, 7*S*-CM, Na^+^]	1292.3	P-1(1292.6), P-2(1292.8), P-3(1292.3)
[DP9, -OEt, 8*S*-CM, H^+^]	1430.3	P-2(1430.5), P-4(1430.8)
[DP9, -OEt, 8*S*-CM, Na^+^]	1452.3	P-2(1452.4), P-3(1452.8)
[DP10, -OEt, 9*S*-CM, H^+^]	1590.3	P-3(1590.9)
[DP10, -OEt, 9*S*-CM, Na^+^]	1612.3	P-4(1612.2)
[DP8, -OEt, 5*S*-CM, H^+^]	1156.2	P-5(1155.4)
[DP8, -OEt, 5*S*-CM, Na^+^]	1178.2	P-5(1178.6)
[DP9, -OEt, 6*S*-CM, H^+^]	1316.3	P-5(1315.5), P-6(1316.6)
[DP9, -OEt, 6*S*-CM, Na^+^]	1338.2	P-5(1338.9), P-6(1338.5)
[DP10, -OEt, 7*S*-CM, H^+^]	1476.3	P-5(1476.5)
[DP10, -OEt, 7*S*-CM, Na^+^]	1498.3	P-5(1497.8), P-6(1498.8)

Experimentally detected *m/z* values close to the theoretically calculated *m/z* (within ±1.0) were shown.

## 4 Discussion

In the present study, we found that the thermal treatment of lysozyme with enzymatically synthesized PLCys induced protein polymerization through SH/SS exchange. The extent and structure of the protein cross-linking can vary depending on pH, because PLCys is soluble in an alkaline (pH >9) solution. In addition, SH/SS exchange is known to be accelerated by enhancing dissociation of SH groups to thiolate anions, and the β-elimination of SS bonds easily occurs under alkaline conditions, resulting in not only reshuffling of SS bonds but also in the production of either lysinoalanine or lanthionine [27], [28]. Indeed, PLCys was unstable as determined by DTNB after it was solubilized in alkaline buffer. Preliminarily, lysozyme reacted with PLCys under an alkaline condition (Na-phosphate, pH 10.0) and aggregated even at 30 °C, though the protein incubated alone or with Cys did not (data not shown). To avoid complicated analyses of the interaction between PLCys and the protein, we explored the possibility of PLCys to cross-link proteins at neutral pH. Although the solubility of PLCys is low at pH 7.0, both thermal treatment and protein denaturation with urea accelerated the PLCys-induced protein polymerization. PLCys peptides involved in the polymer were recovered by reduction with DTT, and subsequent chromatographic analysis elucidated that PLCys played a role as a cross-linker. Jansens *et al.* reported that synthetic additives with multiple SH groups affected the mechanical properties of glassy or rigid wheat gluten materials [29], [30]. Woerdeman *et al.* demonstrated changes in toughness, strain-to-failure, and water absorption in molded gluten specimens made from gluten powder modified with a tri-thiol compound, suggesting that protein networks increased [31]. However, there was no data at the molecular level showing that such multi-thiol materials actually played a role as cross-linkers.

Only a slight amount of PLCys seemed to bind directly to the protein. For quantitative analysis, we tried to label PLCys by mBBr, an SH-specific reagent for fluorometric detection [32], [33]. However, labeled products were noticeably precipitated even when intact PLCys dissolved in DMSO was treated with mBBr. MALDI-TOFMS analysis of the precipitates indicated that not all SH moieties of PLCys were modified (data not shown), in contrast to the complete modification of PLCys by IAM as performed in the present study. Reasons why the S-alkylation of PLCys by mBBr did not succeed might be either steric hindrance or hydrophobicity of the fluorescent tag. Since the structures of fluorescent chromophores are generally large and hydrophobic [34], quantitative analysis of the cross-linked PLCys remains a subject of our research.

The ionization of SH groups is enhanced by the presence of nearby amino groups but oppositely affected by carboxy groups [35], [36]. Thus, the reactivity of SH groups in PLCys may be different depending on their sequence positions. Furthermore, the sensitivity of proteins for the intermolecular SH/SS exchange reaction with PLCys depends not only on the amount of SH and SS but also on their location and micro-environment, such as surrounding or adjacent amino acid residues. Once PLCys bound to a protein, the remaining free SH groups could be stimulated to take part in further SH/SS exchange reactions one after another by their surrounding amino acid residues. This might be why PLCys in the >30 kDa fraction bound to proteins via multiple SS bonds. To support the assumption, detail information on peptide sequences around the SS bonds in protein polymers is necessary. We attempted to investigate whether the disulfide pattern in the polymerized lysozyme was specific or random by means of in-gel digestion for the trimer. The DTT-mediated reduction followed by S-alkylation prior to trypsinization was omitted, because there was no guarantee of the complete S-alkylation of proteins with another reagent other than IAM as described above. However, remarkable *m/z* signals of peptide fragments have not been detected until now. Probably, the precipitation of protein in gel pieces during the washing out denaturant might negatively affect enzymatic cleavage, lowering peptide recoveries. While, other proteins, i.e. bovine serum albumin, ovalbumin, and rice flour proteins, showed various sensitivities for the interaction with PLCys as well as Cys (data not shown). The effects of urea on those proteins were different, although protein denaturation was likely effective for allowing the reactive PLCys to access the target regions of the proteins. Therefore, a mixture of proteins containing either SH or SS would give complexity to PLCys-induced protein cross-linking. Understanding the specific sequence and structure of proteins as well as the reaction condition being important for the cross-linking by PLCys remain to be solved.

In conclusion, enzymatically synthesized PLCys is a promising peptidic cross-linker for proteins. In terms of the availability of PLCys for protein cross-linking, we must overcome a variety of challenges: regulation of the PLCys-induced protein polymerization, analyses of the physicochemical properties and functionalities of the protein polymers cross-linked by PLCys, and investigation of the effects of oxidizing or reducing agents on them. Humans eat many kinds of proteins, including those with cystine structures. Furthermore, it is known that cystine is absorbed in the intestinal epithelium by cystine transporters and is intracellularly reduced to Cys by glutathione [37], [38]. However, for practical application of PLCys in food technology, we should evaluate the safety, digestibility, and potential allergic properties of protein polymers cross-linked by PLCys [39].
