# Supplementary material for: Mechanistic investigation of capability of enzymatically synthesized polycysteine to cross-link proteins
Source: Biochem Biophys Rep. 2016 Jul 21;7:338–46. doi: 10.1016/j.bbrep.2016.07.013 (PMC5613652; doi:10.1016/j.bbrep.2016.07.013)
Supplement: Supplementary file 2 — Supplementary material [file mmc2.docx]

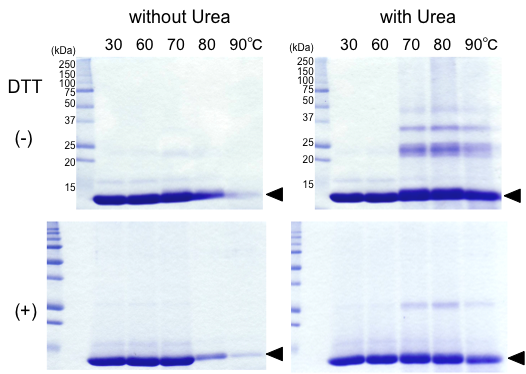


Supplementary 1

Heat-induced aggregation of lysozyme was analyzed by SDS-PAGE. Lysozyme of 3 mg/mL in 100 mM Na-phosphate (pH 7.0) was incubated for 30 min at the indicated temperatures without (left) or with 4M urea (right). After centrifugation of the sample solution, the supernatant was loaded on non-reducing SDS-PAGE (upper, -DTT) and reducing SDS-PAGE (+DTT). These results showed that the protein self-aggregated and precipitated above 70°C.


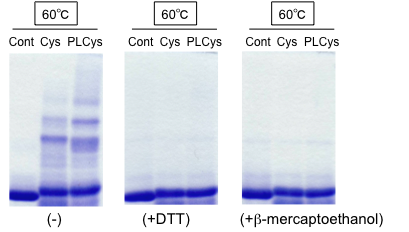


Supplementary 2

Reducing effects of DTT and β-mercaptoethanol on the lysozyme polymers were compared. Lysozyme of 3 mg/mL in 100 mM Na-phosphate (pH 7.0) was incubated for 30 min at 60°C without or with thiol compounds of 10.5 mM DTNB-reactive SH groups. Their supernatants were analyzed by non-reducing (-) and reducing SDS-PAGE. Sample reduction was performed by incubation with 1.5% (w/v) DTT or 3% (w/v) β-mercaptoethanol for 1 h at room temperature in the dark.


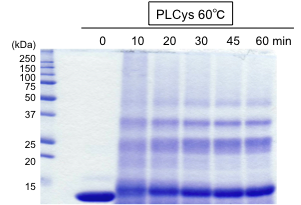


Supplementary 3

Analyses of temporal change of lysozyme during incubation with urea and PLCys at pH 7 by non-reducing SDS-PAGE.
